# Didymin: an orally active citrus flavonoid for targeting neuroblastoma

**DOI:** 10.18632/oncotarget.15204

**Published:** 2017-02-08

**Authors:** Sharad S. Singhal, Sulabh Singhal, Preeti Singhal, Jyotsana Singhal, David Horne, Sanjay Awasthi

**Affiliations:** ^1^ Department of Molecular Medicine, Beckman Research Institute of the City of Hope, Comprehensive Cancer Center and National Medical Center, Duarte, CA, USA; ^2^ University of California at San Diego, La Jolla, San Diego, CA, USA; ^3^ University of Texas Health, San Antonio, TX, USA; ^4^ Texas Tech University Health Sciences Center, Lubbock, TX, USA

**Keywords:** neuroblastoma, p53, didymin, N-Myc, RKIP

## Abstract

Neuroblastoma, a rapidly growing yet treatment responsive cancer, is the third most common cancer of children and the most common solid tumor in infants. Unfortunately, neuroblastoma that has lost p53 function often has a highly treatment-resistant phenotype leading to tragic outcomes. In the context of neuroblastoma, the functions of p53 and MYCN (which is amplified in ~25% of neuroblastomas) are integrally linked because they are mutually transcriptionally regulated, and because they together regulate the catalytic activity of RNA polymerases. Didymin is a citrus-derived natural compound that kills p53 wild-type as well as drug-resistant p53-mutant neuroblastoma cells in culture. In addition, orally administered didymin causes regression of neuroblastoma xenografts in mouse models, without toxicity to non-malignant cells, neural tissues, or neural stem cells. RKIP is a Raf-inhibitory protein that regulates MYCN activation, is transcriptionally upregulated by didymin, and appears to play a key role in the anti-neuroblastoma actions of didymin. In this review, we discuss how didymin overcomes drug-resistance in p53-mutant neuroblastoma through RKIP-mediated inhibition of MYCN and its effects on GRK2, PKCs, Let-7 micro-RNA, and clathrin-dependent endocytosis by Raf-dependent and -independent mechanisms. In addition, we will discuss studies supporting potential clinical impact and translation of didymin as a low cost, safe, and effective oral agent that could change the current treatment paradigm for refractory neuroblastoma.

## INTRODUCTION

Among childhood malignancies, neuroblastomas (NB) constitute the most common solid tumors in infants and the third most common class of pediatric malignancies [1–4]. Most cases of NB are highly curable and chemo-sensitive, but treatments are significantly less effective in NB that have increased MYCN activity (MYCN gene, amplified in 20-25% of NB tumors) [5–11]. Patients with MYCN amplification often have incomplete responses to therapy, which leads to relapse. Furthermore, relapsed NB is frequently deficient in p53 function and highly resistant to apoptosis induced by current therapies [12–14]. This has tragic consequences for children with aggressive drug-resistant NB, including high toxicity from chemotherapeutic regimens and high rates of therapeutic failure. MYCN stimulates while p53 inhibits the expression of multiple cancer stem-cell genes, suggesting that targeting MYCN could overcome the therapy resistant phenotype of recurrent NB [4, 12]. *De-novo* NB is a highly chemotherapy sensitive neoplasm that rarely has p53 mutation suggesting that the ability of amplified MYCN to confer poor prognosis is independent of p53 [13]. In the highly treatment-resistant phenotype, functional loss of p53 amplifies the survival-promoting effects of MYCN [12–14]. Because there are no effective pharmacological agents that directly and specifically inhibit MYCN, agents that can upregulate endogenous inhibitors of MYCN expression or activity are attractive for treating resistant NB.

One possible such agent is didymin, a citrus-derived natural compound that kills NB cells regardless of their p53 functional status and causes regression of NB tumors in xenograft models when given orally [15]. Didymin was not toxic toward either mature neurons or neural stem cells in newborn mice, suggesting its potential safety for use in young children, the group that is most frequently affected by NB. Recently, it was reported that didymin can cause cell death in non-small cell lung cancer (NSCLC) in a p53-independent manner [16]. The use of chemotherapeutic drugs such as cisplatin, doxorubicin and vincristine is highly limited in refractory and relapsed NB because of frequent loss-of-function mutations in the tumor suppressor p53 [17, 18]. Therefore, the identification of signaling pathways through which didymin acts in NB could potentially improve the effective management of NB.

Raf-kinase inhibitory protein (RKIP) is a novel protein that interacts with MAP/ERK kinases and acts as an inhibitor of the MAPK pathway [19]. RKIP is an established metastasis suppressor protein and, given the role of the MAPK pathway in regulating cancer cell survival and metastatic potential, RKIP has been the focus of recent investigations assessing the effects of novel anticancer agents [19–22]. Loss of p53 leads to increased activity of multifunctional proteins, such as RLIP76, which mediate enhanced proliferation, invasion and drug/radiation resistance in NB [23, 24]. In recent studies, we found that RKIP, which also regulates MYCN activation, is transcriptionally upregulated by didymin and appears to play a key role in the anti-NB actions of didymin [15].

RKIP can also inhibit G-protein coupled receptor kinase-2 (GRK2), which activates a number of cancer-critical pathways and broadly regulates ligand-receptor signaling through clathrin-dependent endocytosis (CDE). It can activate MYC through Raf-independent pathways [22, 25–29]. Both RKIP and GRK2 can be regulated by protein kinase C (PKC) isoenzymes that are active in resistant NB [30, 31]. These three kinases, as well as the MYCN-regulated *Let-7* micro-RNA, can broadly regulate down-stream of multiple peptide hormone-ligand interactions by modulating CDE [26, 30–33]. The importance of these pathways in mediating characteristic apoptosis-resistance in p53-mutant NB or the mechanisms of didymin action is not known. In this regard, an effective strategy for targeting NB in genetically identified risk groups should decrease the incidence of NB as well as improve survival after initial diagnosis. Therefore, novel therapeutic strategies to ameliorate the prognosis of NB patients are required.

## DIDYMIN

**Figure 1:**
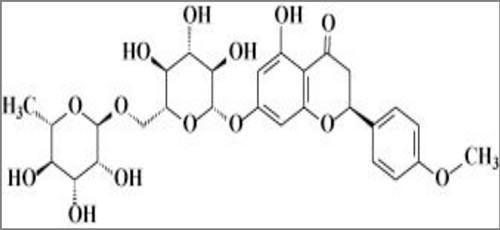
Chemical structure of didymin

**Figure 2:**
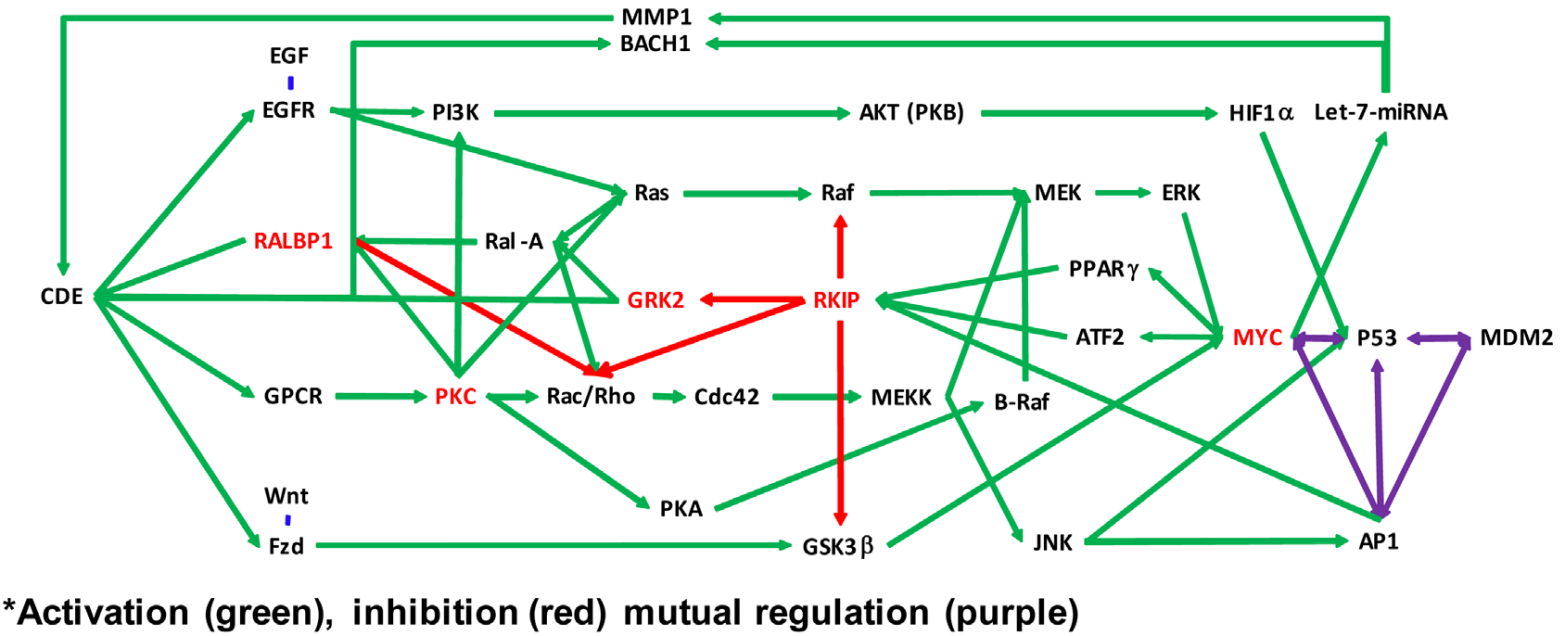
Molecular pathways affected by didymin Schematic showing MYCN down-regulation by didymin and based on network analyses of our proteomic studies that focused on cancer-related pathways active in NB cells. The MAPK/ERK pathway (also known as the Ras-Raf-MEK-ERK pathways) is a cell-signaling pathway that plays a vital role in normal cell division and growth. Mutations that result in constitutive activation of the MAPK/ERK pathway have been implicated in a broad range of solid tumors and are associated with tumor resistance to standard cancer therapies. Inhibition of MEK, which is pivotal protein kinase in the MAPK/ERK pathway, may block growth of solid tumors and interfere with development of resistance. Didymin inhibits MYCN through Raf-dependent and -independent mechanisms by inhibiting CDE through RKIP, GRK, PKC, and the Let-7 micro-RNA.

**Figure 3:**
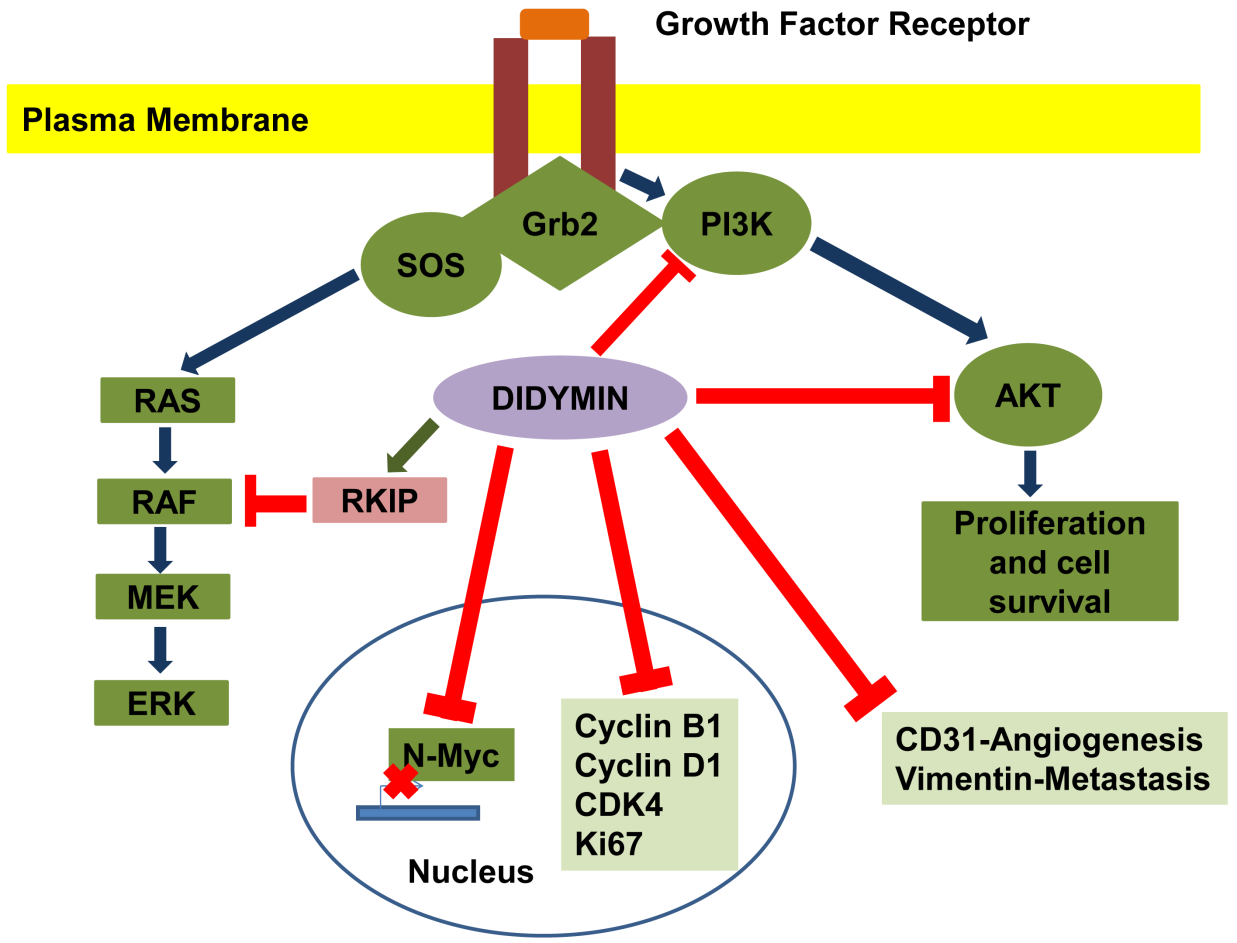
Effect of didymin on neuroblastoma signaling pathways Didymin inhibits N-Myc transcription, down-regulates PI3K, Akt, vimentin, and up-regulates RKIP. Didymin also down-regulates cyclin D1, CDK4 and cyclin B1, which is a down-stream effector of p53 mediated cell-cycle regulation. Didymin decreases expression of the angiogenic marker CD31, proliferation marker Ki67, and N-Myc *in vivo* as revealed by histopathological examination of resected tumors. Blue -Normal signal transduction, Green-Up regulation of signaling protein, Red-Inhibitory effect of didymin.

Didymin is a commercially available (Indofine Chemical Company, Hillsborough, NJ; > 99% pure by HPLC) dietary flavonoid glycoside that is derived from citrus fruits (Figure 1) and has high therapeutic efficacy in NB [15]. Our studies indicate that didymin inhibits cell proliferation and induces apoptosis regardless of p53 status, making it a candidate novel therapy for aggressive, relapsed, or refractory NB. Didymin is orally bioavailable and highly effective, and does not cause significant toxicity in normal tissues, including neural tissue and neural stem cells. We have identified increased expression of RKIP as a key effect of didymin [15]. RKIP directly inhibits key cancer-promoting kinases such as GRK2, Rac, and GSK3β, and these kinases can regulate MYCN expression through Raf-independent mechanisms (Figures 2 and 3). In this review, we discuss the role of these pathways in the therapy-resistant phenotype of p53-null and/or MYCN-amplified NB, the signaling mechanisms that mediate the aggressive and treatment-resistant behavior of refractory NB, the mechanisms of action of didymin, and key *in vivo* data in animal models that can facilitate the clinical translation of didymin in the treatment of NB.

## ANTIOXIDANT EFFECTS OF DIDYMIN

The general structure of flavonoids gives them antioxidant activity through free-radical de-localization to reduce the toxicity of reactive-oxygen species (ROS). In addition, many fragmentation products of flavonoids are substrates or regulators of drug-metabolizing and stress-responsive enzymes that can exert biological antioxidant effects [34–38]. These enzymes include the p450 system, glutathione-linked enzymes, drug-efflux transporters, and associated regulatory proteins. Because PKC, Ras, PI3K, Ral, p53, and MYC are activated by oxidative stress [39–41], generalized suppression of oxidative stress by didymin could reduce the activity or expression of these cancer survival, proliferation, and invasion pathways. Our findings that levels of 4-hydroxynonenal (4HNE), a down-stream marker of oxidative stress, increased in p53-mutated resistant NB cells [23] suggest that generalized oxidative stress as characterized by increased levels of OH·, such as O_2_^−·^, lipid-hydroperoxide radical, mitochondrial membrane potential [42], glutathione levels, catalytic activities of antioxidant enzymes (superoxide-dismutase, catalase, glutathione-peroxidase, glutathione S-transferase) and membrane transporters (Ralbp1, P-glycoprotein, multidrug-resistance associated protein) should also be altered in p53-deficient cells. In addition, oxidative-stress responsive signaling and transcriptional regulatory proteins such as PKC, Ras, PI3K, Ral, p53, and MYC should be increased. We predict that the antioxidant function of didymin will reduce each of these consequences and responses to oxidative stress in a concentration-dependent manner. Given the results of our studies with RKIP [15], we expect that most of the effect of didymin will depend on the presence of functional RKIP. The possibility that RKIP effects depend on reduced activity in the PI3K pathway, could be examined by over-expression of constitutively active Akt mutants. As an antioxidant, didymin should suppress cellular levels of lipid peroxidation (LPO) products, resulting in anti-proliferative and pro-apoptotic signaling and transcriptional programs in NB cells (Figure 3). The antioxidant properties of didymin [43] suggested that it could have anticancer functions: oxidative stress is known to inactivate anti-proliferative pathways, including the tumor suppressor PTEN, and to promote pro-proliferative kinase signaling through the PI3K, MAPK and Akt/mTOR pathways [39]. One possible explanation for the effect of didymin is its chemical structure. This structure could allow didymin to rescue cells from oxidative damage by directly scavenging free radicals through hydrogen atom donation or indirectly by activating antioxidant enzymes. Interestingly, didymin inhibited the growth of NSCLC in a p53-independent manner with its predominant effects mediated by the Fas/Fas L apoptotic pathway [16].

## DIDYMIN INHIBITS MYCN THROUGH RAF-DEPENDENT AND -INDEPENDENT MECHANISMS BY INHIBITING CDE THROUGH RKIP, GRK, PKC, AND LET-7 MICRO-RNA

Because the transcription of RKIP could be regulated by MYCN alone or in concert with PPARγ, AP1, or ATF2, it is possible that the changes in RKIP are a consequence rather than a cause of MYCN depletion. Although this is unlikely because RKIP knockdown partially negates didymin activity, it cannot be ruled out because the effect of Raf inhibition by RKIP was not studied. In addition, RKIP interacting proteins including STC2, PI3KR1, MED10 and TRAF6 are transcriptionally regulated by MYCN and p53, and could modulate RKIP activity. PPARγ has been shown to down-regulate MYCN and regulate cell growth and differentiation in NB cells. The transcription of AP1 and MYC are mutually regulated [44–47]. ATF2 can regulate MYC transcription and *vice versa*, and ATF may also regulate AP1 transcription through activation by PKC.

GRK2 has been shown to be active in NB cell lines [28, 29, 48, 49]. We predict that didymin-mediated RKIP upregulation reverses apoptosis-resistance by inhibiting CDE through effects on GRK2. Other pathways inhibited by RKIP are NFκB, GRK2, Rac, and Wnt. RKIP may inhibit epithelial-to-mesenchymal-transition (EMT) and activate the intrinsic apoptotic pathway by inhibiting NFκB in other cancers [50, 51]. Inhibition of EMT by didymin suggests a possible role of NFκB, but there is little evidence for a significant role of NFκB in NB. GSK3β in the Wnt pathway, known to be active in NB, is mutually regulated by MYC [51–53], but its role in NB or didymin mechanisms is not known. Phosphorylation of RKIP at S^153^ promotes its dimerization, which switches its inhibitory activity from Raf1 to GRK2 [28]. GRK2 activates MYC through the Rac/PAK/MEK/ERK/Myc pathway and may inhibit cell migration induced by didymin. GRK2 also directly activates ligand-receptor pair endocytosis through CDE [29]. Blocking CDE by depleting RLIP76 causes apoptosis in both p53-wildtype and mutated NB and leads to regression of NB xenografts [23]. RLIP76 is a stress-responsive anti-apoptotic glutathione-electrophile conjugate (GS-E) transporter that links the oxidative-stress defenses to CDE and survival through peptide-hormones [39–41]; thus, RKIP inhibition could also mediate apoptosis and inhibit invasion by inhibiting the epidermal growth factor receptor (EGFR) through phosphorylation at S^991^ and Y^998^ [54]. EGFR is over-expressed in NB, activating PI3K to promote cell proliferation in NB [55]; both EGFR and RLIP76 signaling ultimately feed to MYC [39]. The *Let-7* (lethal-7) micro-RNA is another MYC-dependent pathway through which RKIP could inhibit CDE and invasion [32]. *Let-7* and MYC are mutually regulated, and *Let-7* inhibits metastasis through a pathway consisting of BACH1 (a basic region-leucine zipper transcription factor) which is regulated by endocytosis, suppresses p53-mediated cell senescence, and regulates oxidative-stress responses [22, 56]. *Let-7* also regulates MMP1, a pro-angiogenic matrix metalloproteinase that regulates CDE [57]. Epidermal growth factor (EGF) and vascular endothelial growth factor (VEGF) are regulated by *Let-7* [32, 58], but the mechanism underlying their activity and their functional significance in NB is not fully understood. Previous studies provide strong evidence for a mechanistic role of RKIP induction in the actions of didymin, but how the transcription of RKIP is regulated by didymin is not known. We predict that the observed down-regulation of MYCN by didymin will be due to complex feed-back mechanisms that regulate the transcription of MYCN through one or more of the above transcription factors, and that defining these mechanisms could identify novel targeting agents that would be synergistic with or antagonize didymin. The significance and validity of these findings could eventually be tested in human clinical trials.

## TARGETING DIDYMIN TO TREAT P53-NULL NB

Surgical resection of localized NB is effective only in the initial stages; advanced and metastatic NB require aggressive multi-modal treatment with chemotherapy and radiation [59–62]. Although the vast majority of NB are chemosensitive and curable, those with MYCN amplification or p53-mutations are considered high-risk because of their aggressive behavior, tendency to relapse, and resistance to therapy. Children with high-risk NB have a substantially lower cure rate despite treatment with more aggressive and toxic therapies, and even the most promising approaches are limited by side effects during the course of treatment. There is no consensus regarding optimal therapeutic agents for p53-mutant NB. Although overall cure rates are high in NB, the ~20% children with MYCN amplification have a poorer prognosis because of intrinsic or acquired drug-resistance. Although p53 function is almost always normal at diagnosis and the disease is quite sensitive to therapy, tumor cells from recurrent NB are frequently deficient in p53 function due to mutations or deletions. We hypothesize that didymin will offer a highly effective, non-toxic approach to treatment of MYCN amplified and/or p53-null NB that will change the treatment paradigm for this therapy-resistant disease and reduce treatment related toxicity, morbidity and mortality.

## DIDYMIN EXERTS ANTI-NEOPLASTIC EFFECTS *IN VIVO*

Didymin was shown to have *in vivo* efficacy against MYCN-amplified NB xenografts when given by oral gavage at 2 mg/kg body weight on alternate days for sixty days beginning 10 days after tumor inoculations (at which time all animals had palpable tumor ~ 40 mm^2^). Treated mice had significantly smaller tumors as compared to controls, which showed uncontrolled tumor growth. Tumor weight was reduced by more than 50% at 60 days without overt toxicity. The final tumor weight on day 60 was 0.91 g in the didymin-treated group compared to 1.97 g in controls (*p* < 0.001) [15]. These studies demonstrate sustained regression of NB xenografts following treatment with the targeted agent, didymin. Didymin caused no weight loss or significant changes in blood counts or metabolic tests. The serum concentration of didymin 1 h after an oral dose of 2 mg/kg was 2.1 + 0.3 μM, and LC-MS studies revealed a complex mixture of degradation products, characteristic of other flavonoids [63–65]. Histopathological examination of paraffin-embedded tumor xenograft sections revealed lower levels of a proliferation marker (Ki67), an angiogenesis marker (CD31), and MYCN expression in didymin-treated NB xenografts as compared to untreated, which further supports the *in vitro* results [15].

The effectiveness of didymin-targeted therapy compares favorably with other promising targeted agents in NB. Plants have long been an attractive source of novel anticancer drugs. Indeed, approximately 50% of anticancer drugs are natural or semi-synthetic products. Epidemiologic studies provided evidence that citrus consumption is associated with reduced all cancer incidence [66, 67]. *Cirsium setidens* and *Aster scaber* are perennial edible plants that are rich in flavonoids and grow wild in the mountainous regions of Korea. Studies from the Kwon laboratory demonstrated that the neuroprotective effects and mechanism of action of three major phytochemicals (daucosterol, pectolinarin, and astragalin) isolated from edible plants against H_2_O_2_-induced cell death of human NB SK-N-SH cells occurred through down-regulation of MAPK pathways and upregulation of hemeoxygenase, catalase, and superoxide dismutase antioxidant genes. Furthermore, these effects were associated with reduced oxidative stress in SK-N-SH cells *in-vitro*. These cell culture studies have provided important insights but do not prove the *in vivo* efficacy of any tested agent [68]. Citrus bergamia (bergamot) is a small tree belonging to the family Rutaceae. Recently, bergamot juice attracted attention because of its remarkable content of flavonoids [69, 70]. Navarra *et al.* evaluated the effect of bergamot juice *in vitro* and in a spontaneous metastatic NB SCID mouse model. They showed that bergamot juice significantly affected SK-N-SH and LAN-1 cell proliferation and reduced cell adhesiveness and invasion *in vitro*, but failed to reduce primary tumor weight *in vivo* in NB xenograft model [71].

Human malignant NB is characterized by poor differentiation and uncontrolled proliferation of immature neuroblasts. Retinoids at low doses are capable of inducing differentiation, while the flavonoids epigallocatechin-3-gallate and genistein at relatively high dose can induce apoptosis. Das *et al.* used a combination of retinoids and flavonoids to control growth of SH-SY5Y NB cells, and found that combinatorial treatment induced neuronal differentiation concomitant with downregulation of telomerase activity and N-Myc. But this treatment also led to over-expression of neurofilament protein and subsequently increased apoptosis in the differentiated cells. Although these studies suggested that combining a retinoid and a flavonoid worked synergistically to control the growth of NB cells *in-vitro*, the combination was not explored *in vivo* [72]. Thus, targeted therapeutics for NB developed from phytochemicals (daucosterol, pectolinarin, and astragalin), bergamot, or retinoids do not appear to be as effective as didymin in comparable experiments *in vivo*. The greater effectiveness of targeted therapy using didymin can be understood in terms of a chemical as well as biochemical signaling model (Figures 2 and 3).

## RELATIVE EFFICACY OF DIDYMIN

Didymin is a flavone that is common in citrus fruits like oranges, lemons, mandarin and bergamot. Given the absence of any pharmacokinetic data on didymin in the literature, it has been difficult to determine the appropriate dosage of didymin for *in vivo* studies. Therefore, we evaluated didymin at concentrations of 1-20 mg/kg given orally on alternate days for 8 weeks in nude mice bearing NB xenografts. Mice given 2 mg/kg didymin had significantly smaller average tumor volumes when compared to vehicle-treated control mice. All of the didymin-treated mice survived to the end of our study. No signs of toxicity, as judged by parallel monitoring of body weight and tissue sections, were observed in didymin-treated mice [15]. This degree of efficacy and concomitant lack of toxicity compares favorably with other biologically targeted preclinical approaches (Table 1).

**Table 1:** Relative Efficacy of Didymin *in vitro* and *in vivo*

*In vitro* and or/ *in vivo* studies	Target	Didymin Dose	Response	Ref
A549 lung cancer cells	Fas/Fas ligand	6 mg/kg bw, *i.p*. daily for 4 weeks	Inhibited tumor progression	16
HSC-T6 cells and liver tissues	Rat hepatic fibrosis model; ERK/MAPK and PI3K/Akt pathways	0.5 mg/kg bw, *i.p*. daily for 12 weeks	Alleviated liver injury by up-regulating RKIP expression	78
RAW264.7 mouse macrophage cells and liver tissues	Mouse hepatic fibrosis model; MAPK/NFκB pathways	0.5 mg/kg bw, *i.p*. daily for 10 days	Reduced pro-inflammatory cytokines TNFα, IL6, and inhibit NFκB activation *in vivo* and *in vitro*	80
HepG2 liver carcinoma cells	Mitochondrial dysfunction	Range 30 μM to 66 μM	Decreased Bcl-2/Bax ratio and loss of MMP	79
Neuroblastoma SH-SY5Y cells	H_2_O_2_ -induced neurotoxicity	Range 10 μM to 30 μM	Rescue of neuronal cells from oxidative damage.	81
Neuroblastoma SMS-KCNR cells	N-Myc and RKIP	2 mg/kg bw, oral gavage on alternate days for 8 weeks	~55% tumor regression; decreased Ki67, CD31, and N-Myc expression, and increased RKIP expression	15

bw, body weight; *i.p.*, intraperitoneal

RKIP is involved in several cell signaling cascades, and is a specific inhibitor of the MAPK signaling pathway [73]. It directly interacts with both Raf-1 and MEK and disrupts the Raf-1/MEK interaction, thereby preventing MEK activation and its downstream targets [74]. Hung *et al*. demonstrated that didymin has an antiproliferative effect in lung cancer cells. This work was supported by xenograft studies that have shown that didymin delays tumor growth in nude mice, and that the Fas/FasL apoptotic system is involved in didymin-mediated apoptosis [16]. Phthalates, including butyl benzyl phthalate, di-n-butyl phthalate, and di-2-ethylhexyl phthalate, are used as softeners and plasticizers [75]. Epidemiological studies have shown that exposure of breast cancer cells to phthalate esters, may contribute to the progression of cancer by enhancing cell proliferation, migration, and invasion [76]. Didymin, a dietary flavonoid glycoside present in citrus fruits, demonstrates antioxidant and anticancer properties, and Hsu *et al*. revealed that didymin was capable of preventing phthalate ester-associated cancer aggravation [77]. Lin *et al* showed that didymin significantly ameliorated chronic liver injury and collagen deposition and attenuated the mitochondrial membrane potential accompanied by release of cytochrome C. These actions were mediated by inhibition of the ERK/MAPK and PI3K/Akt pathways by regulation of RKIP expression. These results also showed that didymin significantly increased RKIP expression both in liver tissues and HSC-T6 cells (rat hepatic stellate cells) [78, 79]. A follow-up study from the same group revealed that didymin treatment significantly reduced CYP2E1 activity, LPO levels, ROS generation, NO production, and pro-inflammatory cytokines (such as TNFα, IL6, and IL-1β) in liver tissues and RAW264.7 cells (mouse leukemic macrophage cells), but enhanced the activity of hepatic antioxidant enzymes. Further studies showed that didymin significantly inhibited the NFκB and MAPK pathways, representing a protective effect of didymin against CCl_4_-induced hepatotoxicity [80]. Morelli *et al*. showed that the antioxidant property of didymin stimulated superoxide dismutase, catalase, and glutathione peroxidase activity in neuronal cells exposed with H_2_O_2_. This suggests that didymin may be a potential therapeutic molecule for the treatment of neurodegenerative disorders associated with oxidative-stress [81].

## EFFECT OF DIDYMIN IN NORMAL MOUSE BRAIN (NEURO-MORPHOLOGY AND STEM CELL POPULATIONS)

Because the brains of children are still developing, the effects of didymin on the brain must be evaluated before it can be moved forward as a potential therapeutic modality for NB. Singhal *et al* performed a preliminary *in vivo* assessment of didymin's effects in the areas of the brain that have high rates of neural cell proliferation, such as the dentate gyrus, and areas with stem cell populations, such as the subventricular zone. Didymin did not affect neural progenitor cell proliferation or neuronal differentiation rates, as revealed by a lack of any significant changes in expression of Ki67, DCX, or NeuN in the dentate gyrus and the subventricular zone. Normal neuronal tissue expresses low levels of N-Myc and abundant levels of RKIP, a state that is reversed upon oncogenic transformation after which tumor cells display high N-Myc expression and low RKIP expression. In wild-type mice, didymin treatment further increased the expression of tumor suppressor RKIP and decreased N-Myc levels. Therefore it is likely that didymin contributes to a basal protective effect against oncogenic transformation in normal tissue. That didymin had these effects on N-Myc and RKIP expression in normal mice brain without affecting the mature neuronal and neural stem cell population, together with the ability of didymin to exert similar effects in NB xenografts [15], strongly indicate the mechanistic relevance of didymin for both primary and tertiary management of NB. These findings further support that didymin is unlikely to have toxic effects on normal brain tissues, despite lowering N-Myc and increasing RKIP expression.

## STRUCTURE-ACTIVITY-RELATIONSHIP STUDY FOR MECHANISM OF DIDYMIN AND N-MYC INTERACTION

**Figure 4:**
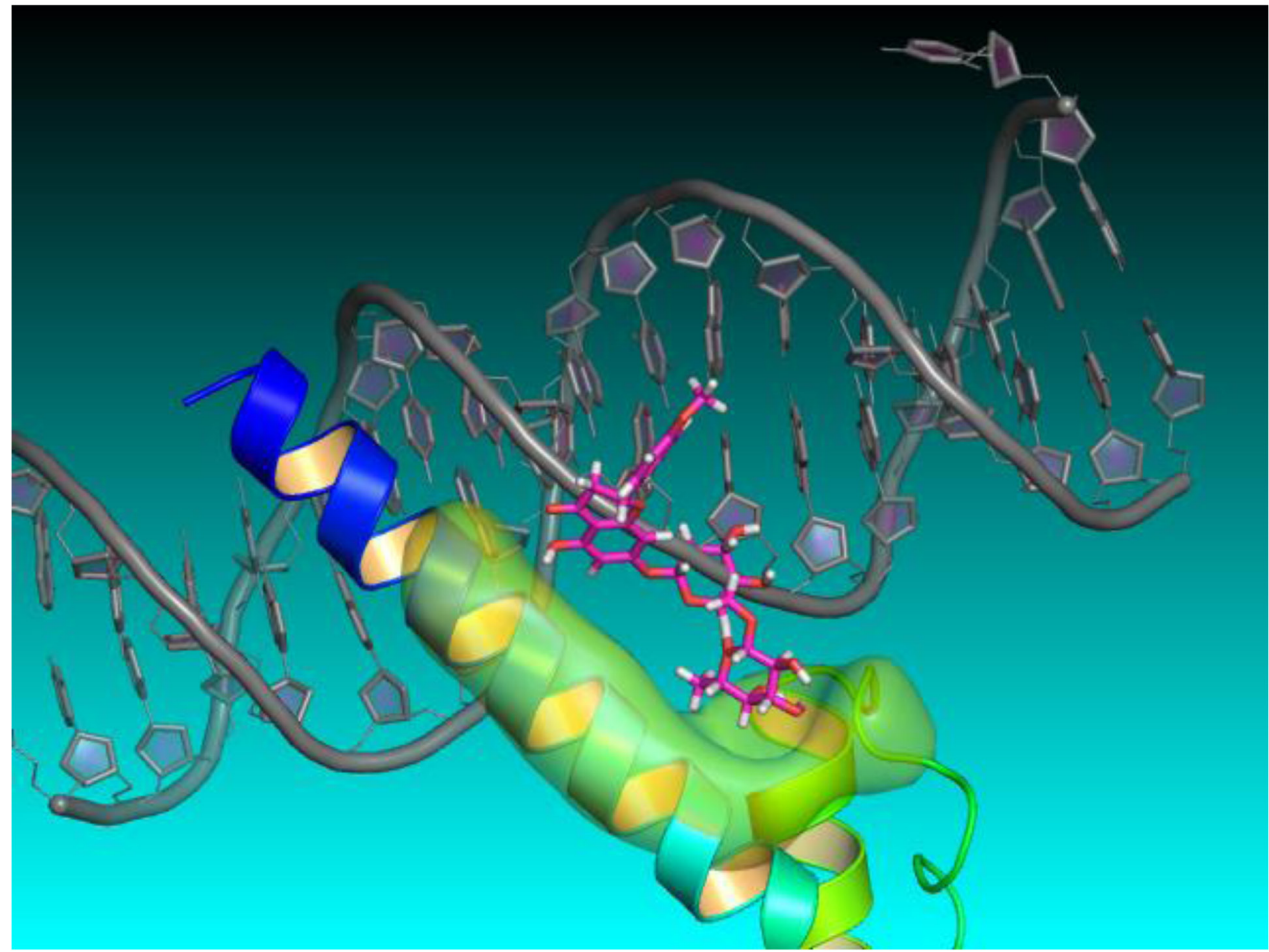
Didymin-NMyc three dimensional ternary homology model Homology structure model of N-Myc derived from protein PDB database using 1NKP, with DNA labeled in grey, N-Myc aa^382-464^ residues labeled in blue, didymin labeled in magenta for carbon, red for oxygen, and white for hydrogen, and the ligand binding surface of N-Myc labeled in green. The model was constructed using Schrödinger Glide docking software and was energy minimized for exploring protein ligand binding mode and mutagenesis validations.

To better understand how didymin might exert its effects on N-Myc-mediated transcription, Singhal *et al* used structure- and ligand-based computer modeling to investigate whether N-Myc interacts directly with didymin. They sought to use a three-dimensional homology model of N-Myc and didymin, generated using the Schrodinger software suite, to validate the active ligand binding mode, based on mutation analysis, in order to better understand the mechanism of action of didymin and to spur rational design for lead optimization (Figure 4).

## DISCUSSION

The causes of NB incidence and progression are topics of intensive investigation. NB is a developmental tumor of young children arising from the embryonic sympathoadrenal lineage of the neural crest, is the primary cause of death from pediatric cancer for children between the ages of one and five years, and accounts for <15% of all deaths from pediatric cancer. Despite recent progress in understanding the biology of NB, there remains a paucity of effective treatments. Important tumor-promoting pathways in NB include activation of N-Myc, loss of the tumor suppressor p53, and cellular acclimatization to elevated levels of oxidative stress [2–4, 14, 36]. Increased oxidative stress leads to generation of ROS and inactivation of phosphatase such as PTEN, which in turn leads to increased activation of intracellular kinase signaling [36, 44–46]. Current treatments for NB have major drawbacks, including lack of effectiveness in p53-mutant tumors, lack of specificity for NB cells, and severe side effects.

We have identified a citrus-derived flavonoid, didymin that addresses each of these drawbacks. Didymin is a palatable dietary flavonoid that is orally active, making it a candidate for convenient oral administration in children. Because didymin is non-bitter, time and effort can be saved in developing an effective means of administration (i.e., it is unlikely taste modifying additives will be needed for oral formulations for children). Instead, it is likely didymin can be administered in milk or other liquid diets or formulations for infants and children, for whom it usually it not feasible to use derivatized preparations such as pills and creams. Our studies have revealed that didymin is highly effective against NB both *in vitro* and in mouse xenografts *in vivo* [15]. Importantly, we have demonstrated that didymin inhibits multiple targets that are critical for driving NB (PI3K, N-Myc and vimentin), but not related targets that are important for neuronal homeostasis such as Sox2, Nestin, DCX, and NeuN. Didymin treatment was tolerable and appears to be non-toxic, as revealed by DCX staining of newly formed neurons, NeuN staining of mature neurons in the hippocampal dentate gyrus, Ki67 staining of proliferating neural progenitors, and Sox-2 staining of neural stem cells in the subventricular zone of brains in control and didymin-treated mice. High-throughput proteomics approaches have identified N-Myc and its regulator, RKIP, as a strong candidate targets for the activity of didymin. N-Myc is a growth-stimulating protein that is typically overexpressed in NB. Therefore, we postulate that didymin exerts its anticancer effects by suppressing N-Myc through RKIP. We also anticipate that RKIP and N-Myc are mechanistically linked to didymin, and didymin will cause both N-Myc dependent and independent inhibition of the growth of NB cells and NB stem cells.

Mechanistically, the inhibition of N-Myc by didymin as shown through protein, mRNA, and promoter-reporter assays was a hallmark of our studies [15]. Amplification and over-expression of N-Myc is associated with both the incidence and refractoriness of NB [82–84]. Hence, the ability of didymin to inhibit N-Myc at both the transcriptional and translational levels reflects its potential utility as a novel interventional strategy for NB. In addition, didymin has both fundamental mechanistic and clinical- translational significance due to its potential to regulate critical nodes of NB signaling that impact the incidence and progression of NB (Figure 3). The over-expression of N-Myc represents a significant enabling molecular event in the incidence and progression of NB, whereas modulation of oxidative-stress impacts the strength of signaling through phosphatases such as PTEN and downstream kinases. Studies by Singhal *et al*. on p53-wildtype and p53-mutant refractory NB have shown that MAP is upregulated in p53-mutant NB, and contributes to the regulation of the toxic effects of ROS and apoptosis-resistance in these cells [23].

The present review collectively provides a strong rationale for the anti-proliferative and pro-apoptotic properties of the novel flavonoid didymin in NB regardless of the p53 status of a given tumor. These studies also provide evidence that didymin is an orally bioavailable non-toxic compound that could fill the need for effective therapies for drug-resistant NB, and provide a generalized method for overcoming resistance mediated by loss of p53 function. These findings have elucidated a role for RKIP in the mechanisms of didymin action, but whether this effect is mediated primarily through Raf-inhibition, the activity of other down-stream pathways, or Rac, GRK2, PKC is not yet known. We believe that the effects of RKIP in these pathways represent multifaceted p53-independent signaling mechanisms that can regulate MYCN through interference with peptide-hormone signaling at the level of CDE. Further studies would provide valuable knowledge regarding the mechanisms through which p53 loss mediates drug-resistance in NB, potential strategies to attack this problem, and crucial information regarding the mechanisms of action, spectrum of *in-vivo* efficacy, and basic pharmacology of didymin.

## CONCLUSIONS

Selecting appropriate compounds or agents for study and development is a major challenge in evaluating natural herbal compounds that may have inherent anticancer properties. We have overcome this challenge by developing *in silico* algorithms for predicting the anticancer activity of natural compounds. Our algorithms are based on the theory that cell cycle and receptor-ligand signaling are linked by the mercapturic acid pathway (MAP), because the rate of endocytosis-dependent internalization of receptor-ligand pairs is directly proportional to the rate of flux of metabolites in the MAP [24]. Our model predicts the rate of flow of metabolites in the MAP according to kinetic constants for the enzymes in the pathway, the concentrations of intermediates, and the inhibitory or stimulatory effects of key signaling nodes. Using this model, we have successfully predicted the anticancer activity of the novel and potent compound, didymin in NB [15]. Our studies revealed that didymin can effectively target NB, which prompted studies on the effects of didymin on critical signaling proteins that regulate cell proliferation, cell cycle progression, and apoptosis in NB. The mechanisms of action of didymin could involve a number of downstream mediators given that didymin has potential anti-oxidant properties [43]. ROS stimulate the activation of critical signaling pathways, including the MAPK pathway [85], and the modulation of ROS can lead to a plethora of effects on cellular proliferation and apoptosis [86, 87].

The differential regulation of RKIP and N-Myc was a salient finding of our study given the opposing roles played by these proteins in the incidence and progression of NB [15, 86, 88]. Silencing of RKIP leads to partial reversal of the survival of NB cells. Given evidence pointing to the prominent role of RKIP in regulating the central axes of proliferative and apoptotic signaling through regulation of Raf, GSK3β, and cyclin D1, studies by Al-Mulla *et al.* are important in directing the study of molecules like didymin that enhance RKIP expression [89–91]. In our studies, we observed upregulation of N-Myc after the knock-down of RKIP in NB [15]. RKIP is an important mediator of the anticancer effects of didymin, but the roles of various effectors of didymin, such as Akt, PI3K, GSK3β, and N-Myc, in RKIP-positive and -negative tumors remains to be established by further studies involving knock-down or over-expression of the respective proteins. Also, future studies are needed to reveal whether the differential regulation of N-Myc consequent to RKIP knock-down is mediated through GSK3β in NB cells. Such studies would benefit the rational development of didymin formulations as well as personalized combinations of anticancer drugs with didymin to achieve better clinical response in patients with differing tumor genotypes and drug-sensitivity profiles. Our findings regarding the novel, safe, and palatable dietary flavonoid didymin have revealed its anticancer properties irrespective of N-Myc amplification and p53 mutation status in NB. Thus, didymin represents a highly promising flavonoid with potential clinical significance to effectively prevent the incidence of NB and as an innovative approach for new treatment strategies proposed by the New Approaches for Neuroblastoma Therapy Consortium.

## RELEVANCE TO HUMAN HEALTH

NB is a prevalent but therapeutically challenging pediatric cancer, accounts for 15 percent of all pediatric cancers, and is the most common extra-cranial solid tumor in children [59]. Amplification of N-Myc plays a role in the pathogenesis of primary NB, whereas acquired mutations of p53 lead to refractory NB. Identification of dietary compounds that can target N-Myc and exert anticancer effects independent of p53 status would be significant in the management of NB. Therefore, we investigated the anticancer properties of didymin in NB. Didymin effectively inhibited proliferation and induced apoptosis irrespective of p53 status in NB. Importantly, didymin inhibited N-Myc, as confirmed at the protein, mRNA, and transcriptional levels by promoter-reporter assays. Our studies also indicate that didymin is absorbed effectively after oral administration [15]. Thus, didymin represents a novel and relevant dietary agent for treatment of NB and in this context, further studies on its mechanisms of action should help to develop and test various didymin doses and formulations for use in children. Success in identifying novel mechanisms for regulating cellular levels of MYCN, delineating the mechanisms of action and of resistance, and eventual clinical deployment of a low cost, safe, and effective oral treatment for drug-resistant NB can change the current treatment paradigm, can help cure children afflicted with this disease, and alleviate treatment related toxicities and stress. Overall, our study of didymin represents a “first of its kind investigation” toward developing an effective interventional strategy for NB [15]. Importantly, didymin is well tolerated by both mature neuronal cells and neural stem cells while being selectively toxic for NB cells, which, along with its inhibitory effect on the oncogene N-Myc and stimulatory effect on the tumor suppressor RKIP makes it an ideal candidate for targeting both primary and tertiary NB management. The palatability, safety, and oral availability of didymin further support its translational anticancer potential. Thus, didymin represents a mechanistically sound flavonoid of specific relevance for further development towards control of human NB.

## FUTURE PERSPECTIVES

NB is the most common childhood solid tumor originating outside the nervous system and the most common tumor in infants. Despite advances in the understanding of the molecular basis of NB, effective interventional strategies have remained out of reach. The amplification of N-Myc plays a primary role in the pathogenesis of NB, and further acquisition of p53 mutations leads to refractory and relapsed NB tumors. Based on a molecular modeling approach, we identified the citrus-derived compound didymin as a potential anti-NB agent. Our studies revealed that the potent anticancer properties of didymin are mediated by down-regulation of N-Myc and up-regulation of the metastasis suppressor RKIP. The effects do not depend on p53 status of the tumor cell, indicating potential broad efficacy of didymin [15]. Furthermore, these studies indicate that didymin is safe, with no measurable effect on neurogenesis in the normal brain. The palatability of didymin as compared to other flavonoids, which are often bitter, together with its ability to be absorbed following oral administration, further support its potential translational impact as an anticancer agent. Our further studies will evaluate the mechanism by which didymin blocks the growth of NB, determine the optimal dosing conditions, and further test its efficacy against clinically relevant spontaneous NB tumor models. These key studies will set the stage for clinical translation of didymin, which has the potential to significantly lower the morbidity and mortality associated with NB and potentially to have utility against other cancers.

Loss of p53 function and increased cellular MYCN are frequent characteristics of recurring NB that play a pivotal role in mediating drug-resistance. Our further studies will answer questions regarding the mechanisms underlying this remarkable effect using state-of- the-art technology to increase knowledge of cellular mechanisms that regulate MYCN function in NB, and begin to define the breadth of applicability in treatment as well as secondary prevention of NB. Although didymin itself represents a particularly innovative therapeutic angle, it could also be easily combined with traditional therapies as an adjuvant. If successful, this palatable non-toxic natural compound could be delivered on a long-term basis or to susceptible populations. This type of approach would fundamentally change the management of NB and potentially open the door to preventative strategies in high-risk populations.

## HIGHLIGHTS

Didymin is a novel, non-toxic, and highly relevant citrus flavonoid that has potential utility for effectively targeting NB in children as it can prevent the growth of NB that has both N-Myc amplification and mutant p53.

Didymin is not bitter, in contrast to most other flavonoids. This gives great flexibility in developing formulations that would be easy to orally administer to children.

Didymin upregulates RKIP levels and inhibits N-Myc at the protein, mRNA, and transcriptional level. Thus, the anticancer potential of didymin likely arises from its multi-specific anticancer mechanism of action.

Overall, didymin appears to be a palatable, well-tolerated, and effective alternative to conventional treatment approaches for NB.

## Abbreviations

CDE: clathrin-dependent endocytosis
GSH: glutathione
GRK: G-protein coupled receptor kinase
GS-E: glutathione electrophile conjugates
GST: glutathione S-transferase
4HNE: 4-hydroxy nonenal
MAP: mercapturic acid pathway
MMP: mitochondrial membrane potential
NB: neuroblastoma
PARP: Poly ADP ribose polymerase
PI3K: phosphatidylinositol 3-kinase
PKC: protein kinase C
RKIP: Raf-kinase inhibitory protein
RLIP76: Ral-interacting protein
ROS: reactive-oxygen-species
VEGF: vascular endothelial growth factor
